# Long-Term Follow-Up of Right Ventricle to Pulmonary Artery Biologic Valved Conduits Used in Pediatric Congenital Heart Surgery

**DOI:** 10.1007/s00246-022-02956-3

**Published:** 2022-07-03

**Authors:** Michael J. Lewis, Torsten Malm, Anna Hallbergson, Fredrik Nilsson, Jens Johansson Ramgren, Kiet Tran, Petru Liuba

**Affiliations:** 1 Divisions of Cardiac Surgery, Pediatric Heart Center, University Hospital, S-221 85 Lund, Sweden; 2 Divisions of Cardiology, Pediatric Heart Center, University Hospital, Lund, Sweden; 3 Divisions of Tissue Bank, Pediatric Heart Center, University Hospital, Lund, Sweden; 4 Department of Clinical Science, Lund University, Lund, Sweden; 5 Department of Dermatology, Skåne University Hospital, Landskrona, Sweden

**Keywords:** RV-PA valved conduit, Pulmonary homograft, Aortic homograft, Bovine jugular vein graft

## Abstract

Valved conduit reconstruction between the right ventricle (RV) and the pulmonary circulation is often necessary in the surgical treatment of complex congenital heart defects. The aim of this study is to evaluate the long-term performance of the three types of conduits we have used and assess risk factors for conduit failure. Retrospective, single-center review of 455 consecutive pediatric patients with 625 conduits from 1990 to 2019 undergoing RV-to-pulmonary artery (PA) reconstruction with a valved conduit. The three conduit types investigated were pulmonary homograft, aorta homograft, and bovine jugular vein (BJV) graft. Overall patient survival was 91.4%, freedom from conduit replacement (FCR) was 47.4%, and freedom from reintervention (FFR) was 37.8% with a median follow-up of 8.7 years (interquartile range 4.3–13.3 years). For pulmonary homografts, 10-, 20-, and 28-year FCR was 79.6%, 68.6%, and 66.0%, respectively. For aortic homografts, 10-, 20-, and 30-year FCR was 49.8%, 31.5%, and 23.0%, respectively. For BJV grafts, 10- and 19-year FCR was 68.1% and 46.0%, respectively. When controlling for baseline variables, FCR was similar for pulmonary homografts and BJV grafts. Overall patient survival was excellent. Risk factors for conduit failure in patients operated with reconstruction of the RV-PA outflow tract included low age, low weight, small conduit size, and certain cardiac diagnoses. There was no evidence for a shorter life span of the second graft. Pulmonary homografts and BJV grafts performed similarly but the risk of endocarditis was greater in the BJV group.

## Introduction

Homografts—both aortic and pulmonary—have been used for more than 50 years in the reconstruction of continuity between the subpulmonary ventricle and the pulmonary circulation [1–5]. Non-homograft biologic conduits, particularly bovine jugular vein (BJV) grafts, have gained popularity in recent years [6–11]. Conduit availability, cost, and performance all play important roles in surgical decision making.

Pediatric heart surgery in Sweden was centralized to two centers in 1994, with each center ultimately responsible for roughly half of the country’s pediatric heart surgery needs. The tissue bank in Lund—which opened in 1985—processed, cryopreserved, and supplied all homografts used in this study. As such, we had detailed knowledge of the homografts we used in a representative population of Sweden’s inhabitants. We used pulmonary and aortic homografts throughout the study period, though aortic homografts were used more restrictively later in the study period; BJV grafts (Contegra, Medtronic, Minneapolis, MN, USA) were used starting in 2003.

Studies have demonstrated higher freedom from conduit replacement (FCR) or failure with pulmonary homografts compared to aortic homografts [1–3, 12]. Similar studies were often limited to short- [13] or intermediate-term follow-up [1–3, 12, 14], had a heterogeneous group of patient ages [4], did not include non-homograft biologic conduits [2, 3, 12–17], or had high rates of conduit failure [6] or mortality [5]. More contemporary reports have compared the use of BJV grafts to cryopreserved homografts [3, 6–11], present long-term-follow-up [4], are multi-institutional [6, 9, 18] and assess the need for reintervention or conduit exchanges [4, 14, 17–19]. The aim of our study was to investigate the long-term performance of the biologic conduits we use in right ventricle (RV)-to-pulmonary artery (PA) reconstruction for common congenital heart defects in children. We assessed—both for our entire patient population and a statistically similar subgroup—FCR, freedom from reintervention (FFR), incidence of endocarditis, and risk factors for conduit failure.

## Materials and Methods

### Patients

All patients who received an RV-to-PA conduit were retrospectively reviewed for the period January 1, 1990 to December 31, 2019. Patients with a two-ventricle circulation were included, while those with single-ventricle physiology were excluded. Additionally, patients were excluded from the study if it was not possible to accurately ascertain any of the study endpoints, listed below.

### Study Design

The Ethical Review Board (ERB) at Lund University granted approval for this study (Dnr 2017/133). Patients included were collected through independent registries maintained by the Divisions of Pediatric Cardiac Surgery and Tissue Bank in Lund. Patients were further cross-checked by an operative implant registry maintained by the Division of Critical Care in Lund. Perioperative clinical and imaging data were obtained from both the electronic and archived medical records.

Study endpoints were overall survival, FCR, and FFR, measured from the time of operation through December 31, 2019. FCR was defined as the length of time from operative conduit placement to either operative conduit replacement or patient death. FFR was defined as freedom from either operative conduit replacement (operative conduit intervention other than conduit replacement was not performed) or transcatheter conduit intervention. Number of cardiac operations were noted in an individual patient; these included not only conduit operations but all other operations pertaining to the palliation and correction of the child’s cardiac defect (aortopulmonary shunts and transannular patching, for example). Incidence of endocarditis was assessed in accordance with criteria in the modified Duke classification system [20]. Only cases of endocarditis requiring operative conduit replacement were recorded.

### Conduits

All homografts were collected, processed (including treatment with antibiotics), cryopreserved, and stored at the Tissue Bank in Lund, Sweden. All homografts were sized at the time of processing with Hegar dilators, and again at the time of operation. BJV grafts (Contegra, Medtronic, Inc., Minneapolis, MN, USA) were procured from a single distributor. All operations were performed at our hospital. Anatomic versus non-anatomic choice of conduit placement was decided at the time of surgery, and standard intraoperative protocols were followed with respect to preparation of either cryopreserved homografts or BJV grafts. In the majority of cases, the implanted conduits were moderately oversized (conduit *Z*-score from 0 to + 2). No attempts were made at prospectively matching homografts with patients for blood type.

### Operative Indications and Techniques

While specific thresholds may have changed throughout the duration of the study, decisions regarding primary operation, reoperation or reintervention were made on an individual basis in a multidisciplinary conference. Currently, indications include a combination of decreased physical capacity, progressive RV dilatation, RV end diastolic volume > 150 ml/m^2^ body surface area, RV ejection fraction < 45%, pulmonary/conduit stenosis with peak gradient > 50 mmHg, pulmonary/conduit regurgitation fraction > 40%, tricuspid regurgitation or ventricular arrhythmias.

The operative technique used to correct each patient’s cardiac anomaly varied according to diagnosis. However, the RV-to-PA conduit reconstruction was consistently performed with either single- or double-venous cannulation, aortic arterial cannulation, and a beating heart that was either warm or mildly hypothermic. An adequate runoff was ensured to the pulmonary circulation, and the distal anastomosis was fashioned first. The conduit was trimmed so that the valve was placed as near as possible to the pulmonary bifurcation to avoid compression and deformation by the sternum. The operation was then completed with the proximal anastomosis between the conduit and RV. Pulmonary artery size at the time of operation was assessed and compared to published nomograms [21]. A PA was deemed narrow if either the right or left PA was more than two standard deviations smaller than predicted for patient’s weight. Conduit *Z*-score was defined as the corresponding pulmonary valve size calculated in accordance with patient morphological variables [22]. All homografts were trimmed of excess musculature to avoid calcification and stenosis. A proximal extension was occasionally needed, particularly for homografts: aortic homografts were often extended with the graft’s own ascending aorta, while pulmonary homografts were often extended using either a patch from the graft itself or a separate piece of Dacron. If the conduit’s proximal anastomosis was fashioned at the patient’s native pulmonary annulus, its position was deemed ‘anatomic’. Otherwise, it was deemed ‘non-anatomic.’ Patients received routine perioperative antibiotics. BJV graft patients received aspirin for three months postoperatively.

### Follow-Up

Patient survival status was verified with the national population registry. Reoperations performed on patients after initial RV-to-PA conduit placement were ascertained through either the Swedish national congenital heart disease database (SWEDCON) or independent registries maintained by the Divisions of Pediatric or Adult Cardiac Surgery in Lund. Reinterventions in the form of cardiac catheterizations were ascertained through both the Swedish national health care registry and a registry maintained by the Division of Pediatric Cardiology in Lund.

### Data Analysis

Data were reported as counts, percentages, means, medians, and interquartile ranges. *P*-values < 0.05 were used to ascribe statistical significance. Between-group differences were assessed by one-way ANOVA and Mann–Whitney tests for quantitative variables and Fisher’s exact test for qualitative variables. Survival analyses were produced using the Kaplan–Meier method; between-group differences were assessed using the log-rank sums test.

A subgroup of patients based on conduit type was selected by manually selecting them based on similar baseline variables. Similarity was confirmed, and survival analysis was performed comparing all three conduit types. A further-refined subgroup (pulmonary homografts and BJV grafts) was selected for matched pairs of patients with similar baseline variables using a propensity score. Clinical variables predictive of conduit type were first evaluated by univariable logistic regression. All variables with a *P*-value < 0.15 which were not correlated with other variables were entered into a similar multiple variable logistic regression model. The significant predictive variables were then used to produce a propensity score. A nearest neighbor 1:1 matching technique within a caliper width of 0.1 was used to select matched pairs of patients.

Univariable Cox regression analysis was performed to investigate risk factors for conduit failure. Variables with a *P*-value < 0.2 which were not correlated with other variables were included in the multiple variable Cox regression analysis. Missing data were analyzed and imputated as needed. An appropriate model was selected after multiple regression models were fitted using a retrograde, stepwise approach. Statistical analyses were performed using SPSS version 24 software (IBM SPSS Statistics, IBM Corporation, Chicago, Illinois, USA).

## Results

Throughout the 30-year study period, there were 475 patients who received 647 RV-PA conduits. Twenty patients (4.2%, with 22 conduits, 3.4%) failed to meet the inclusion criteria and were excluded from the study. The remaining 455 patients (625 conduits) are included in this study (Tables 1 and 2). The most common diagnoses included tetralogy of Fallot (TOF, 26.6%), pulmonary atresia with ventricular septal defect (PA/VSD, 23.3%), and truncus arteriosus (TA, 16.9%). Ten were congenitally corrected transposition of the great artery (CCTGA) patients with the morphologic left ventricle serving as the subpulmonary ventricle. In the propensity score-matched group, 184 conduits (92 matched pairs, 131 patients) were selected for comparison (Tables 1 and 3). Follow-up was 100% with respect to overall survival, FCR, and FFR, with a median follow-up of 8.7 years (interquartile range 4.3–13.3 years). Follow-up was longer for homografts than for BJV grafts, as their use was initiated in 2003. Overall survival was 91.4%.

**Table 1:** Diagnostic groups, by conduit number

Diagnosis	Conduit number	Entire group		Propensity score-matched subgroup	
		Patients, *N* (%)	Conduits, *N* (%)	Patients, *N* (%)	Conduits, *N* (%)
Total		455 (100.0)	625 (100.0)	131 (100.0)	184 (100.0)
PA/VSD		106 (23.3)	146 (25.6)	29 (22.1)	35 (19.0)
	1st conduit		100		29
	2nd conduit		41		5
	3rd or 4th conduit		5		1
TOF		121 (26.6)	141 (22.8)	35 (26.7)	43 (23.4)
	1st conduit		118		34
	2nd conduit		22		9
	3rd or 4th conduit		1		0
TA		77 (16.9)	134 (21.5)	26 (19.8)	44 (23.9)
	1st conduit		65		24
	2nd conduit		47		15
	3rd or 4th conduit		22		5
TGA/VSD/PS		33 (7.3)	59 (9.4)	5 (3.8)	15 (8.2)
	1st conduit		33		5
	2nd conduit		18		5
	3rd or 4th conduit		8		5
PS, PI, PA/IVS		33 (7.3)	37 (5.7)	8 (6.1)	10 (5.4)
	1st conduit		31		8
	2nd conduit		6		2
	3rd or 4th conduit		0		0
AS, AI		32 (7.0)	35 (5.4)	8 (6.1)	10 (5.4)
	1st conduit		32		8
	2nd conduit		3		2
	3rd or 4th conduit		0		0
All others ^a^		53 (11.6)	73 (9.6)	20 (15.3)	27 (14.7)
	1st conduit		51		20
	2nd conduit		20		6
	3rd or 4th conduit		2		1

^a^Included diagnoses: ALCAPA, CCTGA, DORV, IAA, TGA

*ALCAPA* anomalous origin of left coronary artery from pulmonary artery, *AI* aortic insufficiency, *AS* aortic stenosis, *CCTGA* congenitally corrected transposition of the great arteries, *DORV* double-outlet right ventricle, *IAA* interrupted aortic arch, *IVS* intact ventricular septum, *PA* pulmonary atresia, *PI* pulmonary insufficiency, *PS* pulmonary stenosis, *TA* truncus arteriosus, *TGA* transposition of the great arteries, *TOF* tetralogy of Fallot, *VSD* ventricular septal defect

**Table 2:** Baseline patient characteristics by conduit number and conduit type, all patients

Conduit number	Conduit type			Mean age at operation ± SD		Mean weight at operation ± SD		Male		Mean conduit diameter ± SD	
		*N*	%	y	*P*-value	kg	*P*-value	%	*P*-value	mm	*P*-value
Total		625	100.0	6.4 ± 5.8	–	23.3 ± 19.4	–	54.9	–	18.0 ± 4.0	–
1st conduit		430	68.8	5.2 ± 5.6	–	19.8 ± 18.8	–	54.2	–	17.2 ± 4.2	–
	Aortic homograft	109	25.3	2.3 ± 3.2	< 0.001	9.5 ± 6.9	< 0.001	53.2	0.85	15.0 ± 3.0	< 0.001
	Pulmonary homograft	190	44.2	8.7 ± 5.8		30.9 ± 20.9		55.8		19.9 ± 4.3	
	BJV graft	131	30.5	2.6 ± 4.0		11.8 ± 11.7		52.7		15.3 ± 2.5	
2nd conduit		157	25.1	8.2 ± 5.1	–	27.7 ± 17.6	–	54.8	–	19.3 ± 2.6	–
	Aortic homograft	33	21.0	6.6 ± 4.0	0.002	22.8 ± 11.5	0.023	51.5	0.58	18.4 ± 1.6	< 0.001
	Pulmonary homograft	74	47.2	9.7 ± 4.7		31.8 ± 17.9		59.5		20.8 ± 2.7	
	BJV graft	50	31.8	7.0 ± 5.7		24.6 ± 18.9		50.0		17.7 ± 1.8	
3rd or 4th conduit		38	6.1	12.6 ± 3.8	–	45.3 ± 15.7	–	63.2	–	21.1 ± 2.2	–
	Aortic homograft	3	7.9	10.2 ± 6.6	0.47	44.0 ± 1.4	0.96	100.0	0.20	19.3 ± 2.1	0.001
	Pulmonary homograft	24	63.2	13.0 ± 3.2		45.9 ± 15.3		33.3		22.1 ± 2.0	
	BJV graft	11	28.9	12.3 ± 4.2		44.4 ± 18.5		54.5		19.5 ± 1.3	

*SD* standard deviation

**Table 3:** Baseline patient characteristics by conduit number and conduit type, propensity score-matched subgroup

Conduit number	Conduit type			Mean age at operation ± SD		Mean weight at operation ± SD		Male		Mean conduit diameter ± SD	
		*N*	%	y	*P*-value	kg	*P*-value	%	*P*-value	mm	*P*-value
Total		184	100.0	5.2 ± 5.2	–	18.8 ± 15.7	–	57.1	–	17.1 ± 3.4	–
1st conduit		128	69.6	4.1 ± 4.9	–	16.0 ± 15.2	–	57.0	–	16.4 ± 3.7	–
	Pulmonary homograft	64	50.0	4.1 ± 4.7	0.67	16.0 ± 15.3	0.95	56.3	1.00	16.8 ± 4.3	0.50
	BJV graft	64	50.0	4.2 ± 5.1		16.0 ± 15.2		57.8		16.1 ± 3.0	
2nd conduit		44	23.9	6.5 ± 4.7	–	21.1 ± 12.8	–	56.8	–	18.2 ± 1.8	–
	Pulmonary homograft	22	50.0	6.3 ± 4.5	0.98	21.2 ± 12.9	0.90	68.2	0.22	18.6 ± 1.8	0.09
	BJV graft	22	50.0	6.6 ± 5.0		21.0 ± 13.0		45.5		17.8 ± 1.7	
3rd or 4th conduit		12	6.9	12.3 ± 2.7	–	40.4 ± 12.8	–	58.3	–	20.2 ± 1.0	–
	Pulmonary homograft	6	50.0	11.9 ± 2.6	0.60	40.0 ± 13.3	1.00	66.7	1.00	20.7 ± 1.0	0.09
	BJV graft	6	50.0	12.6 ± 3.0		40.9 ± 13.6		50.0		19.7 ± 0.8	

*SD* standard deviation

### Freedom from Conduit Replacement

Initial conduit implantation was performed in 430 patients (68.8%), with 195 conduit exchanges (31.2%) performed; 157 (25.1%) were second conduits, and 38 (6.1%) were third or fourth conduits (Table 2). Of the 625 RV-to-PA conduits used, 288 (46.1%) were pulmonary homografts, 145 (23.2%) were aortic homografts, and 192 were BJV grafts (30.7%).

**Fig. 1:**
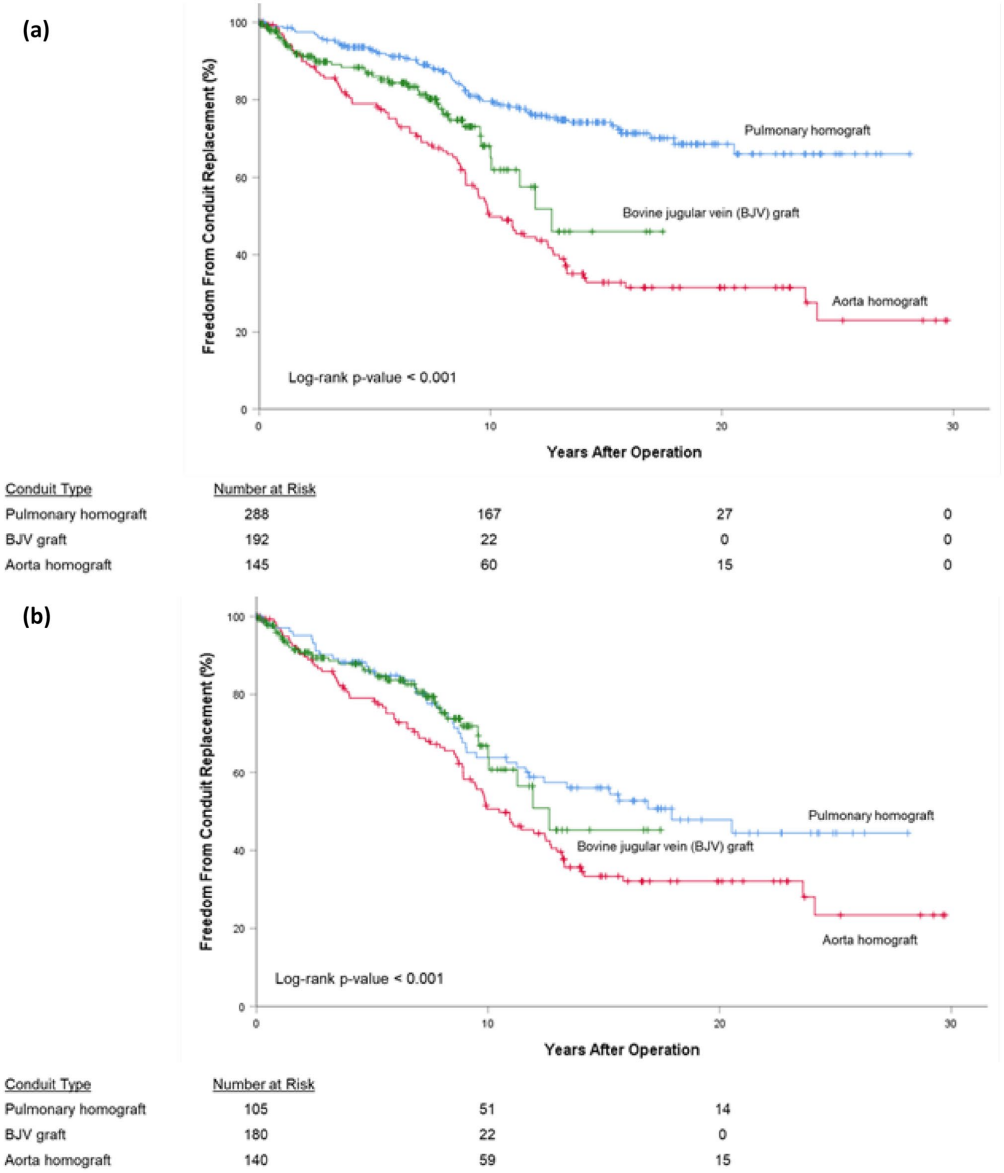

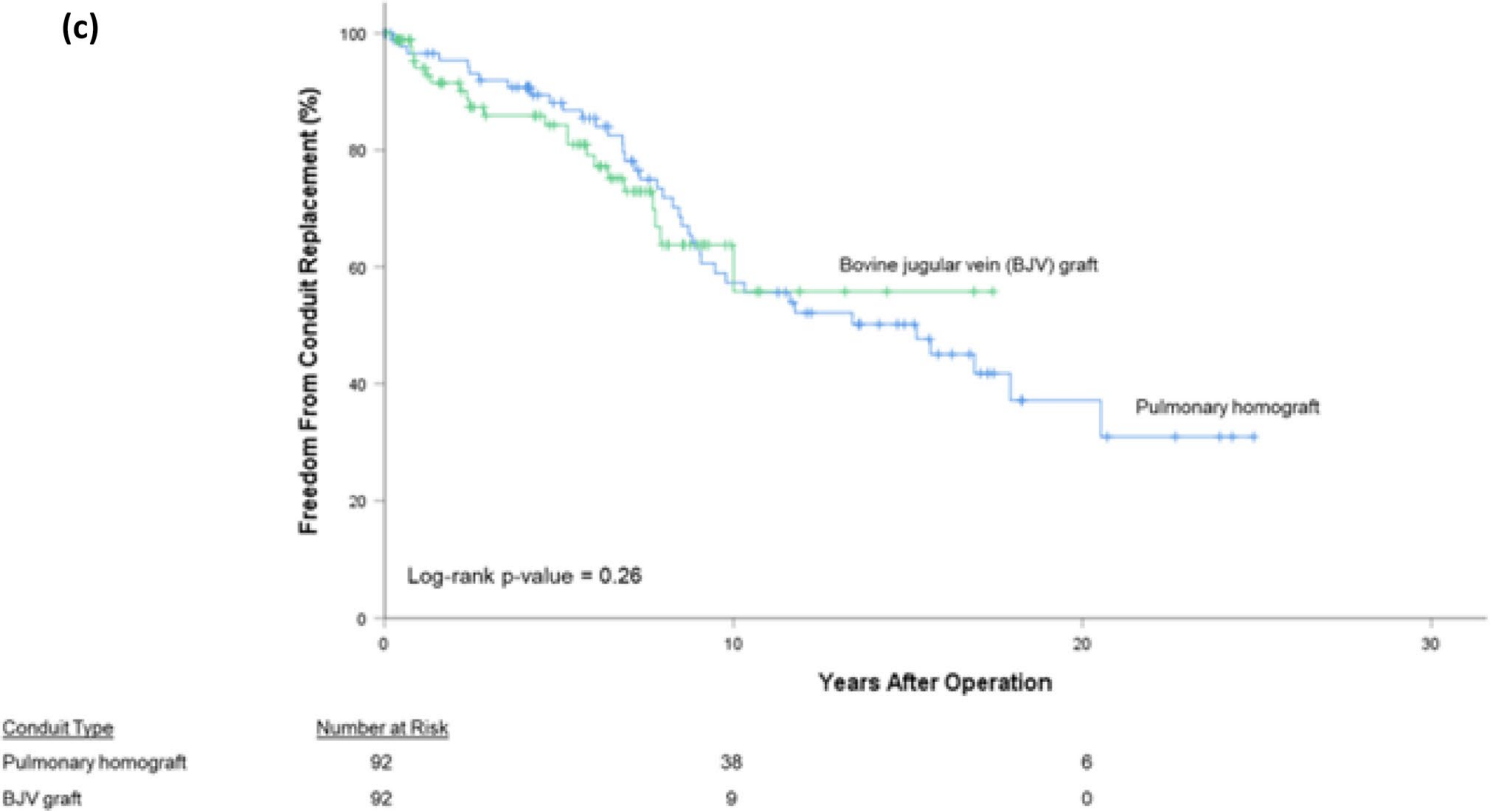
Freedom from conduit replacement (FCR), by conduit type; (**a**) all patients, (**b**) manually adjusted subgroup, (**c**) propensity score-matched subgroup

**Fig. 2:**
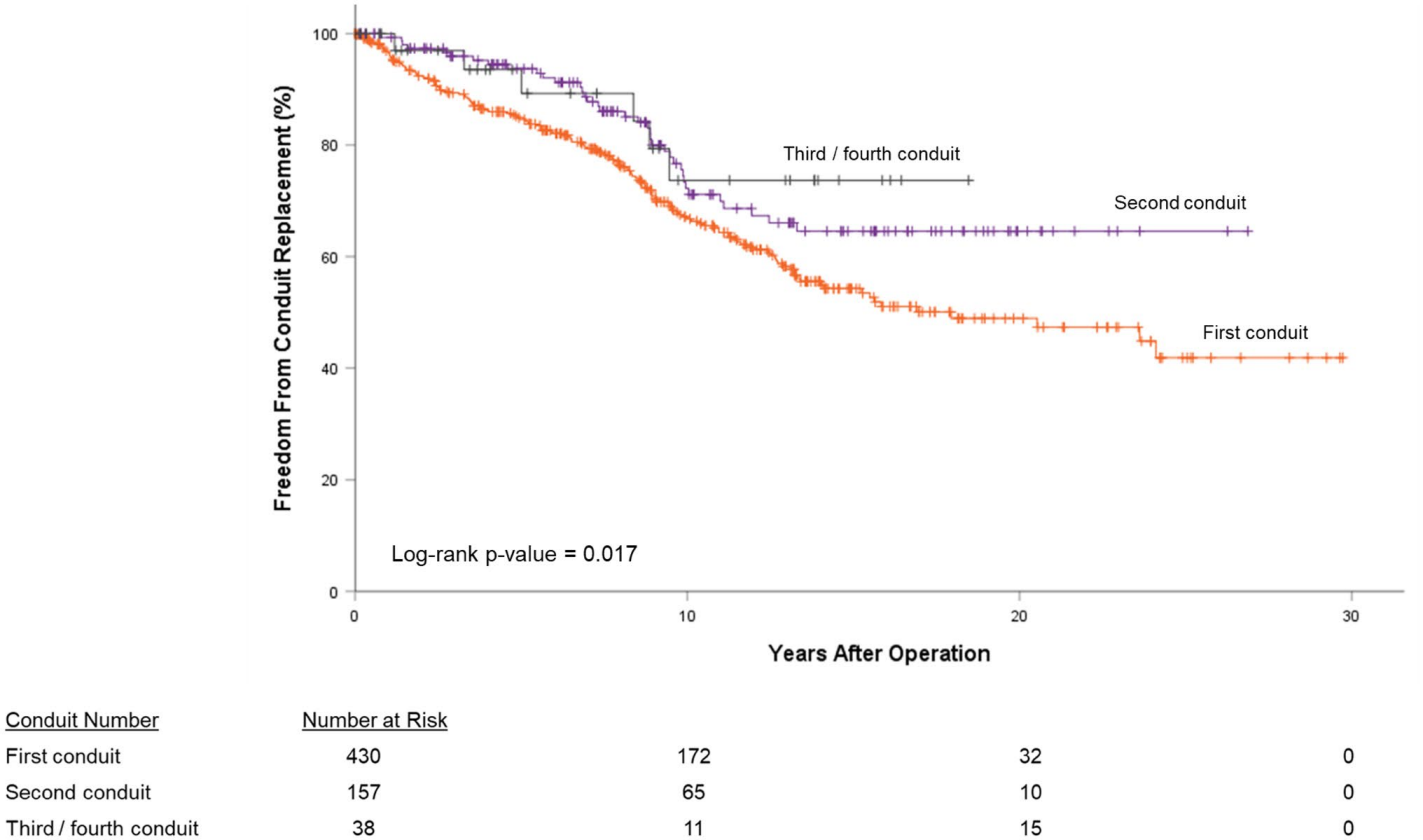
Freedom from conduit replacement (FCR), by conduit number, all patients

For all conduits, 10-, 20-, and 30-year FCR was 68.8%, 54.1%, and 47.4%, respectively. For pulmonary homografts, 10-, 20-, and 28-year FCR (*P*-value < 0.05) was 79.6%, 68.6%, and 66.0%, respectively. For aortic homografts, 10-, 20-, and 30-year FCR was 49.8%, 31.5%, and 23.0%, respectively. For BJV grafts, 10- and 19-year FCR was 68.1% and 46.0%, respectively (Fig. 1a). By diagnostic groups, overall FCR (*P*-value < 0.05) was highest for the Ross-operated aortic valve stenosis/aortic valve insufficiency (AS/AI) group (87.5% at 25 years) followed by TOF patients (72.5% at 29 years). TA patients exhibited the worst FCR (0.0% at 24 years). Third or fourth conduits exhibited the best overall FCR (72.0% at 18 years, *P*-value < 0.05), followed by second conduits (64.6% at 27 years), followed by first conduits (41.9% at 29 years, Fig. 2).

Patients were stratified according to conduit type; a subgroup was selected based on a manual selection of similar baseline variables. The survival curves (Fig. 1b) were similar in appearance to those in Fig. 1a (all patients), again showing different rates of FCR based on conduit type. However, the curves for pulmonary homograft and BJV graft appeared quite similar, prompting further analysis. Propensity score-matched pairs were selected for pulmonary homograft and BJV patients. For this subgroup, overall 25-year FCR was 36.6%. FCR was similar between pulmonary homografts and BJV grafts (*P*-value = 0.26, Fig. 1c).

### Freedom from Reintervention

**Fig. 3:**
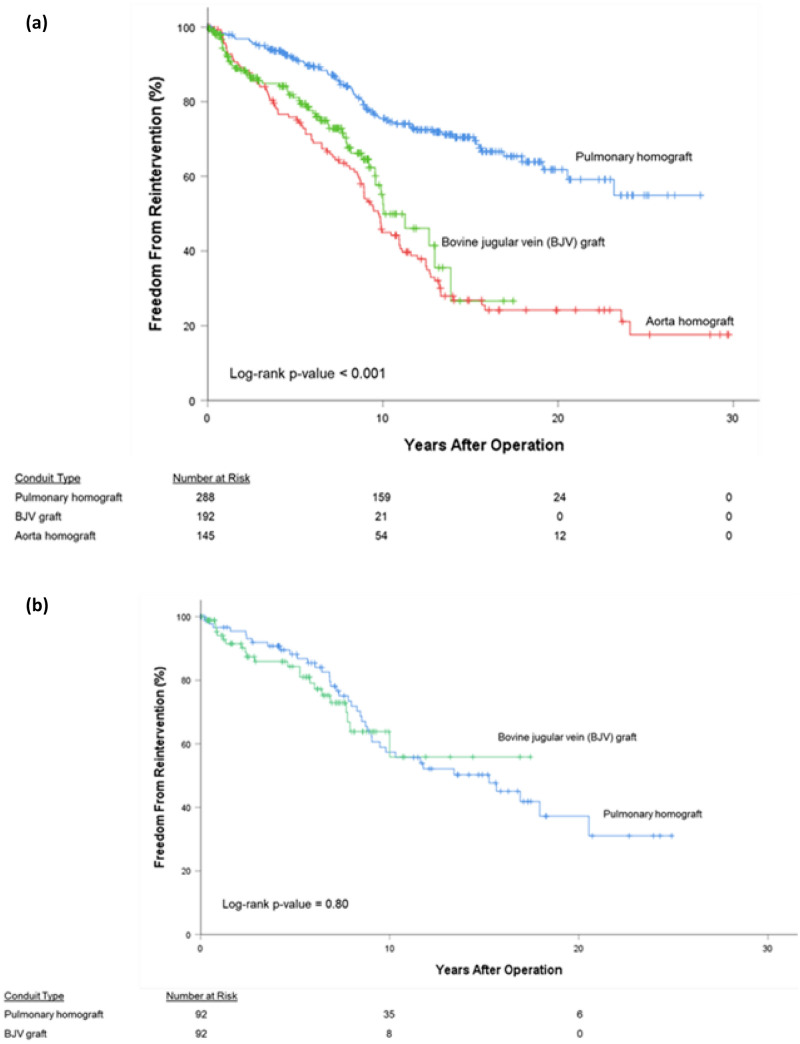
Freedom from reintervention (FFR), by conduit type; (**a**) all patients, (**b**) propensity score-matched subgroup

**Fig. 4:**
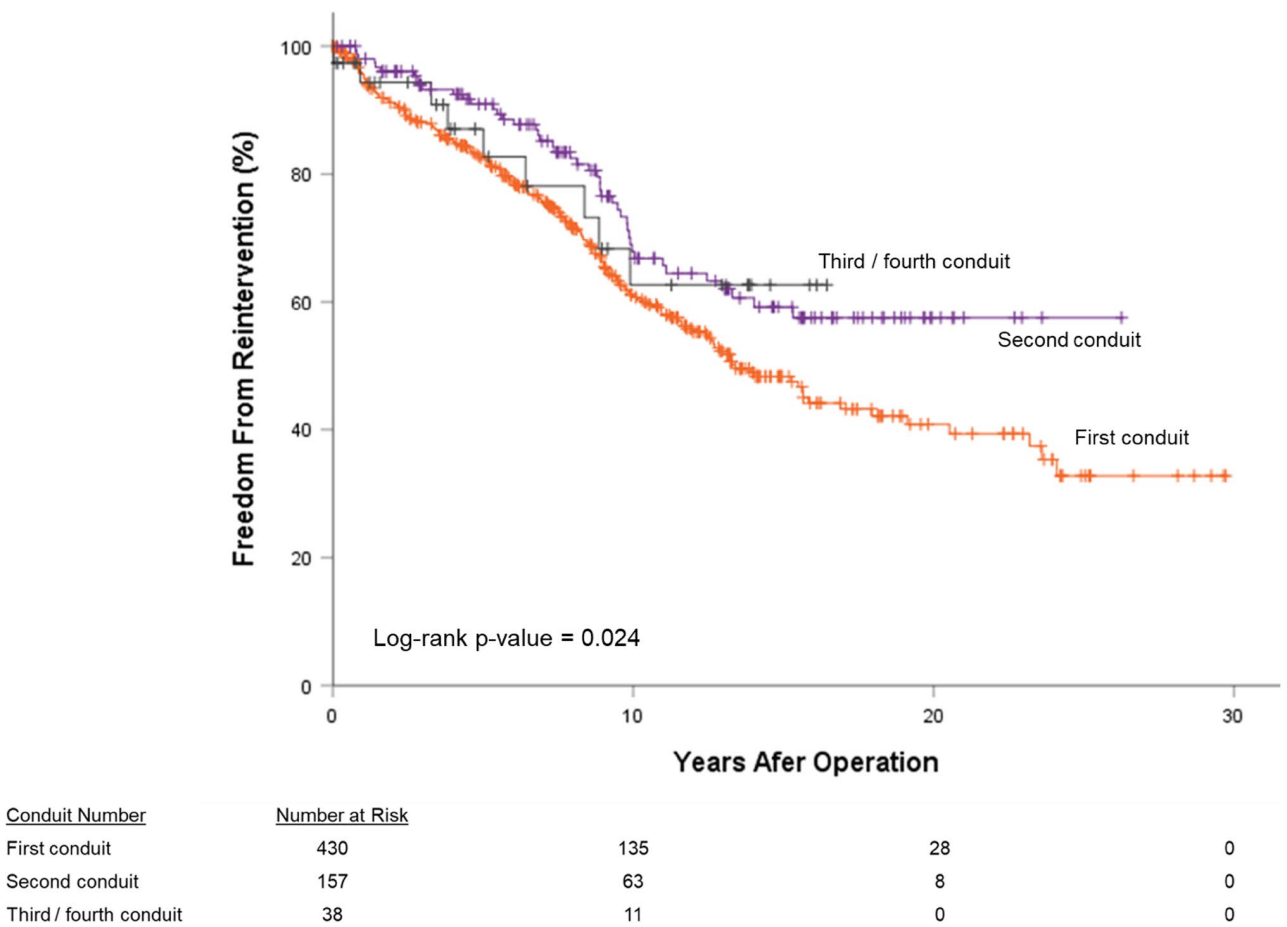
Freedom from reintervention (FFR) by conduit number, all patients

In addition to the 195 conduit exchanges performed, transcatheter interventions were performed in 49 (7.8%) conduits. Whichever event came first—transcatheter intervention or conduit exchange—was measured as the censured event for FFR. For all patients, FFR was 37.8% at 30 years: 54.9% at 28 years for pulmonary homografts, 17.6% at 30 years for aortic homografts, and 26.6% at 17 years for BJV grafts (*P*-value < 0.05, Fig. 3a). By diagnostic groups, FFR was best (*P*-value < 0.05) for the Ross-operated AS/AI group (72.7% at 25 years) followed by TOF patients (59.3% at 28 years). TA patients exhibited the worst FFR (0.0% at 24 years). Third or fourth conduits (62.6% at 16 years) and second conduits (57.5% at 26 years) exhibited similar FFR; first conduits (32.8% at 30 years) exhibited worse FFR (*P*-value < 0.05, Fig. 4. For the propensity score-matched subgroup, overall 25-year FFR was 31.6%. There was no difference in FFR between pulmonary homografts and BJV grafts (*P*-value = 0.80, Fig. 3b).

### Risk Factors for Conduit Failure

Multiple patient-, operative-, and conduit-specific variables were assessed with respect to conduit survival (Table 4). Univariable Cox regression analysis showed significant association with need for conduit replacement for all variables except for gender, presence of at least one narrow pulmonary artery after conduit placement, and previous placement of a BJV graft. Notably, 44 patients (7.0%) had at least one narrow branch PA at the time of correction, yet this did not prove to be a significant factor for conduit failure. The multivariable Cox regression showed the following variables to be significantly associated with need for conduit replacement: smaller conduit diameter (correlated with young patients and low weight), conduits other than second conduits, certain diagnoses (TA and transposition of the great arteries with ventricular septal defect and pulmonary stenosis, TGA/VSD/PS), increasing number of cardiac operations, and conduit placement which occurred during a patient’s first cardiac surgery.

**Table 4:** Cox regression analyses, all patients

Variable	Univariable		Multivariable	
	HR (95% CI)	*P*-value	HR (95% CI)	*P*-value
Gender				
Female	Reference			
Male	1.15 (0.86–1.51)	0.35		
Weight (kg)	0.95 (0.93–0.96)	< 0.001	^a^	
Age at operation (y)	0.87 (0.84–0.89)	< 0.001	^a^	
Conduit type				
Pulmonary homograft	Reference		Reference	
Aorta homograft	3.17 (2.30–4.37)	< 0.001	1.43 (0.95–2.16)	0.09
BJV graft	2.04 (1.36–3.05)	0.001	1.27 (0.79–2.04)	0.33
Conduit number				
First	Reference		Reference	
Second	0.63 (0.44–0.90)	0.01	0.49 (0.28–0.85)	0.01
Third/Fourth	0.53 (0.23–1.20)	0.13	0.29 (0.08–1.00)	> 0.05
Diagnosis				
PA/VSD	Reference		Reference	
TOF	0.48 (0.29–0.78)	0.003	1.44 (0.50–4.17)	0.50
TA	1.91 (1.31–2.79)	0.001	2.17 (1.24–3.83)	0.01
TGA/VSD/PS	1.87 (1.17–3.00)	0.009	3.31 (1.88–5.82)	< 0.001
PS, PI, PA/IVS	0.36 (0.14–0.90)	0.03	1.61 (0.40–6.44)	0.50
AS, AI	0.28 (0.10–0.77)	0.01	1.26 (0.32–5.04)	0.74
All others ^d^	1.11 (0.68–1.80)	0.67	1.91 (1.10–3.30)	0.02
Previous cardiac operation	0.35 (0.26–0.48)	< 0.001	0.48 (0.28–0.83)	0.01
Number of previous cardiac operations	1.76 (1.54–2.01)	< 0.001	2.32 (1.88–2.87)	< 0.001
Conduit diameter (mm)	0.80 (0.77–0.83)	< 0.001	0.87 (0.80–0.95)	0.001
Graft Z-Score	1.28 (1.09–1.52)	0.003	1.02 (0.83–1.25)	0.84
RV systolic pressure after repair (mmHg)	1.01 (1.00–1.02)	0.02	0.99 (0.98–1.01)	0.33
Narrow pulmonary artery	1.29 (0.81–2.05)	0.28		
Proximal hood	2.15 (1.60–2.89)	< 0.001	0.87 (0.56–1.34)	0.53
Proximal hood type			^b^	
Ascending aorta	Reference			
Pulmonary homograft patch	0.45 (0.24–0.85)	0.01		
BJV graft patch	0.58 (0.21–1.60)	0.29		
PTFE patch	0.43 (0.16–1.18)	0.10		
Autologous pericardial patch	2.02 (0.92–4.44)	0.08		
Dacron patch	0.69 (0.40–1.18)	0.18		
Bovine pericardial patch	0.00 (0.00-inf)	0.96		
Anatomic graft position	0.31 (0.22–0.45)	< 0.001	1.11 (0.42–2.90)	0.83
Previous homograft	0.61 (0.42–0.89)	0.01	^c^	
Previous BJV graft	0.71 (0.26–1.93)	0.51		

^a^Correlated with conduit diameter

^b^Removed due to small number of operations (*n* = 199)

^c^Correlated with conduit number

^d^Included diagnoses: ALCAPA, CCTGA, DORV, IAA, TGA

*ALCAPA* anomalous origin of left coronary artery from pulmonary artery, *AI* aortic insufficiency, *AS* aortic stenosis, BJV bovine jugular vein, *CCTGA* congenitally corrected transposition of the great arteries, *DORV* double-outlet right ventricle, *IAA* interrupted aortic arch, *IVS* intact ventricular septum, *PA* pulmonary atresia, *PI* pulmonary insufficiency, *PS* pulmonary stenosis, *PTFE* polytetrafluoroethylene, *TA* truncus arteriosus, *TGA* transposition of the great arteries, *TOF* tetralogy of Fallot, *VSD* ventricular septal defect

### Conduit Replacement due to Endocarditis

For all patients, 18 of 625 conduits (2.9%) were replaced due to conduit endocarditis: four of 288 (1.4%) pulmonary homografts, five of 145 aortic homografts (3.4%), and nine of 192 (4.7%) BJV grafts. There was a significant difference between rates of endocarditis in pulmonary homografts and BJV grafts (*P*-value = 0.04). First conduits (*P*-value < 0.05) required replacement in six of 430 (1.4%) conduits, second conduits nine of 157 (5.7%) conduits, and third or fourth conduits three of 38 (7.9%) conduits.

## Discussion

In this study, we have reported follow-up of up to 30 years of patients operated on with a biologic conduit placed between the RV and the pulmonary circulation. We account for roughly half the congenital heart surgery needs of Sweden, while procuring all homograft conduits from the Tissue Bank in Lund. We sought to compare the performance of the three types of conduits (pulmonary homograft, aortic homograft, and BJV grafts) used at our institution as measured by FCR and FFR. We further assessed for differences in conduit performance by evaluating subgroups of our patients. We investigated factors for conduit failure and assessed for incidence of endocarditis requiring surgical conduit exchange.

Our patients were similar to those commonly described in the literature [1–4, 7, 12, 14, 15, 23, 24], with the largest groups consisting of TOF, PA/VSD, and TA patients. Our 10-year patient survival was 95.3%, comparable to other similar studies [3, 9, 10, 17, 19, 25], which have quoted rates of 10-year survival of between 87 and 96%. Our 30-year patient survival was 91.4%, which was favorable compared with 83% 20-year survival reported by Falchetti and colleagues [8]. There were differences in patient groups with respect to type of conduit inserted, reflecting institutional treatment bias with respect to conduit selection. Our preference has been to use pulmonary homografts when available in an appropriate size, with aortic homografts early in the study period and later BJV grafts as a second choice when appropriate pulmonary homografts were lacking. This can be seen in Table 2, where pulmonary homograft is the dominant conduit of choice throughout the listed subgroups (conduit number).

Overall FCR for the entire group was 47.4% at 30 years and 33.3% at 29 years for the propensity score-matched subgroup. FCR was 68.8% at 10 years and 57.8% at 15 years (whole group) and 58.7% at 10 years and 44.0% at 15 years (subgroup). This compares favorably with similar studies which report 10-year FCR between 30 and 82% [3, 4, 8, 14, 17] and 15-year FCR between 31 and 43% [1, 14].

For the entire group FCR was highest for pulmonary homografts, followed by BJV grafts, and aortic homografts. This superior performance of pulmonary homografts versus aortic homografts reflects the experience of many centers [1, 2, 12, 16]. The superior performance of pulmonary homografts versus BJV grafts has been reported [11], while other centers have reported different findings (see below). Ross-operated patients exhibited the best FCR, consistent with many other studies [13, 16, 17, 25], likely reflecting at the very least the later age and larger conduit size at the time of operative conduit implantation for these patients. TOF patients exhibited similar FCR, likely also explained by conduit placement later in life. This premise holds true as the survival curves for the propensity score-matched subgroup are demonstrated. The pattern seen is a decrease of the apparent benefit of pulmonary homografts as older, larger patients who received larger conduits were removed from the analysis of the subgroup. Various centers have reported either the superiority [9] or equivalence [8, 10, 26] of BJV grafts to pulmonary homografts. Indeed, in the propensity score-matched subgroup, FCR is equivalent between pulmonary homografts and BJV grafts. This was further validated in our multivariable regression analysis, which showed equivalency among conduit types.

Survival curve analysis among first, second, and third/fourth conduits showed worse performance of first conduits compared with subsequent conduit replacements. This was expected, as initial conduits are invariably smaller-diameter and implanted in young, small patients bound to outgrow them. Somewhat surprisingly, this pattern persisted in the multivariable analysis, though the difference between first and third conduits was not quite statistically significant (*P* = 0.051). That this pattern persists after controlling for the effects of other variables suggests that a more thorough explanation is needed. Additionally, we found that conduits placed at a patient’s first cardiac surgery fared worse than those placed after a prior cardiac operation had been performed. These findings suggest a possible inflammatory reaction, both early in a patient’s life and operative course, as proposed by several others [2, 5, 12, 14, 19]. An assessment of a patient’s markers of inflammation throughout their operative course would be potentially enlightening.

Patterns of FFR reflected those seen with FCR. Overall FFR for the entire group was 37.8% at 30 years and 31.6% at 25 years for the subgroup. Our 10-yr FFR was 62.9% (all patients) and 58.0% (subgroup), similar to reported rates of 10-yr FFR of 45–80% [14, 27]. For the entire cohort, FFR was best for pulmonary homografts, followed by BJV grafts, and aortic homografts. Similar to the FCR curves, FFR for the propensity score-adjusted subgroup is equivalent between pulmonary homografts and BJV grafts. Indeed, other centers have reported similar rates of FFR between pulmonary homografts and BJV grafts [10].

Smaller conduit diameter (correlated with patient age and weight at time of operation) was associated with need for conduit replacement in our multivariable model. This reflects similar findings from multiple studies [3, 6, 11–13, 17, 18, 28, 29] in which small conduits implanted in young, small patients are those associated with earlier conduit failure. Again, patient outgrowth and the cumulative effects of an inflammatory reaction could be significant contributing processes. An increasing number of implanted homografts has been suspected to be a risk factor for earlier homograft deterioration [1, 12, 14], as anti-homograft antibodies could sensitize a recipient towards future homograft degeneration. We indeed found that increasing number of prior cardiac operations was a risk factor for earlier conduit degeneration. Prior BJV conduit was not found to be a risk factor (univariable analysis), while prior homograft was correlated with conduit number, and was not included in the multivariable analysis, despite being found to be a risk factor on the univariable analysis. While certainly interesting, a quantification of patient immune response was outside the scope of our study. In any case, consideration towards timing of and ultimate number of interventions is warranted.

Certain diagnoses were found to be more at risk for earlier conduit replacement in our multivariable analysis. Compared with PA/VSD patients, both TA and TGA/VSD/PS patients exhibited worse conduit survival. A smaller, heterogenous group of diagnoses was also shown to have worse FCR, though this was not significant on univariable analysis, and the number of patients in each diagnostic group were too small to reach meaningful conclusions. Anatomic graft position [11, 17, 26] has been investigated as a variable for conduit deterioration. The results of our multivariable analysis failed to show that non-anatomic graft position was related to earlier conduit deterioration. What is particular about certain diagnoses that lead to differences in FCR is clearly not fully elucidated by the other variables which we chose to study. Further assessment of such factors, such as conduit length, angularity, or compression by adjacent structures, for example, could prove useful.

As stated earlier, we used predominantly moderately oversized (conduit *Z*-score from 0 to + 2) conduits. Within the range we used, we found no association between conduit *Z*-score and conduit performance. An RV systolic pressure of less than half the systemic systolic pressure was achieved in almost all cases, except for cases in which, for instance, a VSD was left open or fenestrated. Within our range of RV pressure after conduit insertion, we found no association between it and conduit performance. Similarly, no association was found between a narrow PA and conduit performance, as nearly all cases of conduit insertion were predicated on ensuring runoff to adequately sized pulmonary arteries.

Reported rates of endocarditis (including medically treated cases) in BJV grafts have been reported in the literature: 5.4% [30] to 11.3% [31], with BJV grafts representing a nine-fold increase in the risk of endocarditis [20] compared with homografts. In a recent multicenter trial evaluating transcatheter pulmonary valve replacement inside of previously placed BJV conduits, the risk for valve endocarditis was 4.3% [32]. Our risk for conduit endocarditis requiring conduit exchange (i.e., excluding medically treated cases) was 2.9% for all patients: 4.7% for BJV conduits, 3.4% in aortic homografts, and 1.4% for pulmonary homografts. However, as seen in the FCR analysis of our subgroup, the higher incidence of endocarditis in BJV grafts did not lead to a greater frequency of conduit failure compared with pulmonary homograft. We also observed an increasing incidence of endocarditis requiring conduit replacement with increasing conduit number. Closer study would be needed to see whether this phenomenon represents something more than a marker of the risks inherent in multiple reoperations.

The limitations to this study include its single-center, retrospective nature and the inevitable selection and treatment biases which accompany such studies. We had a clear bias with respect to type of conduit implanted as all patient subgroups were different. We did not analyze era of treatment, despite evolving conduit preferences and thresholds for intervention. We accounted only for cases of conduit endocarditis which required surgical conduit exchange, thereby not accounting for those that were treated medically. While we tried to capture as many clinically relevant variables as possible, there are several known (anatomical, immunologic, etc.,) and unknown variables that we did not include in our analysis. Furthermore, any statistically significant differences we have shown relating to conduit survival may not align themselves with the clinical realities of patient anatomy, conduit availability, and surgical judgement.

The need for RV-to-PA reconstruction is common in congenital heart surgery, as is the need to replace and reintervene on previously placed conduits. The two conduits we currently use most frequently are pulmonary homografts and BJV grafts. Irrespective of conduit type, small conduits implanted in small patients most reliably require earlier replacement. However, assuming operations can be performed in similar patients with similar conduit sizes, pulmonary homograft and BJV graft perform equally well, either as primary conduits or conduit replacements. Aortic homografts have a shorter life span but can be used when no other graft is available or under special circumstances. Second conduits perform no worse than, and indeed better than, first conduits. Pulmonary homograft was less susceptible to endocarditis requiring conduit exchange compared to BJV graft. The findings of this study could have direct clinical implications for pre- and intra-operative decisions for patients expected to require repeated RV-PA conduits during their lifespan.

## Abbreviations

AS/AI: Aortic valve stenosis/aortic valve insufficiency
BJV: Bovine jugular vein
CCTGA: Congenitally corrected transposition of the great arteries
FCR: Freedom from conduit replacement
FFR: Freedom from reintervention
PA: Pulmonary artery
PA/VSD: Pulmonary atresia with ventricular septal defect
RV: Right ventricle
TA: Truncus arteriosus
TGA/VSD/PS: Transposition of the great arteries with ventricular septal defect and pulmonary stenosis
TOF: Tetralogy of Fallot
